# On Returning Inflation to Target

**DOI:** 10.1007/s10272-022-1035-8

**Published:** 2022-04-08

**Authors:** Catherine L. Mann, Lennart Brandt

**Affiliations:** Bank of England, Bartholomew Lane, EC2R 8AH London, UK

**Keywords:** E31, E52

## Abstract

To gauge the breadth of current inflation and prospects for inflation returning to target, we consider disaggregated measures of CPI infl ation to evaluate trends and then consider diff erent scenarios for the realisation for wages and prices.

In the latter half of 2021, inflation in the United Kingdom as measured in the Consumer Price Index (CPI) surged, more than doubling from 2% in July to 5.5%, for the 12 months to January 2022. Survey data on price and wage developments tell the micro story. The most recent summary of business conditions by the Bank of England's agents reported that pay awards in 2021 were 2.5%-3.5%, with some awards of 5%-7% (Bank of England, 2022a). Firms in the latest Decision Maker Panel (DMP) survey reported price increases of 5.4% on average for the three months to February (Bank of England, 2022b). This momentum from prices and wages is pushing up expectations for 2022, with agents reporting expected pay settlement of 4.8%[1] and firms expecting price increases of some 4.5% for 2022. If realised, headline inflation could stay strong for longer, well into 2023, particularly if exacerbated by the geopolitical events of early 2022.

Before assessing the prospects for returning inflation to the 2% target, and the role for monetary policy, it is important to review the sectoral sources of the 2021 inflation surge. First, going into the pandemic, the UK's CPI price level was roughly trending along its 2% inflation path, unlike in the US or the euro area, where inflation had been persistently below target. Second, global demand recovery and supply limitations, as well as geographical shipping mismatches in 2021 yielded robust inflation momentum, particularly for energy and core goods – all of which are mostly external to the UK economy. However, a domestic supply-demand imbalance has also been apparent in the UK with production constraints, shortages of lorry drivers, and other widespread recruiting difficulties leading to domestic cost-push pressures above and beyond external sources.

Will the underpinnings to the global dynamics and their domestic equivalents moderate, with inflation easing? Or will the dynamics of 2021 repeat in 2022 to keep inflation strong for longer? To gauge the breadth of current inflation and prospects for inflation returning to target, we consider disaggregated measures of CPI inflation to evaluate trends and then consider different scenarios for the realisation for wages and prices.

With regard to the first consideration, while it may have been true initially that inflation was mostly a phenomenon of external shocks generating price increases in a few volatile components of the CPI, going into 2022 this story no longer holds up. With regard to the second consideration, if expectations are realised, wage and price dynamics may become embedded into contracts beyond 2022, making for a self-reinforcing inflation path. It matters for forward-looking monetary policy whether underlying inflation is broad or narrow and whether firms and workers expect to recoup the costs incurred in 2021 in their wage and price contracts of 2022 and beyond.

## Measuring underlying inflation

The Bank of England's Monetary Policy Committee, and most other major central banks, sets its policy in order to achieve price stability which is defined in terms of a certain aggregate price index, which in the Bank's case is the Consumer Price Index. Naturally, a variety of shocks will disturb the trajectory of this index at any point in time, which blurs the signal from any one data point. The Bank's remit (HM Treasury, 2021) recognises that optimal monetary policy may look through some disturbances which knock the inflation rate off target in the short term, so long as in the longer-term trend, price growth is anchored at 2%. Responding to every up-and-down move in the CPI would whip-saw monetary policy, potentially causing instability in financial markets and introducing unnecessary variation in the broader macroeconomy. However, this raises the question of how to estimate trend growth or so-called underlying inflation.

Attempts to measure underlying inflation can be broadly split into two categories: exclusion-based indices, which reflect inflation only in some parts of the overall basket; and estimation-based measures, which use some type of statistical model to extract the underlying signal from the

1 See Chart 3.9 in the February 2022 Monetary Policy Report (Bank of England, 2022c).

* This article is based on a speech at the Official Monetary and Financial Institutions Forum on 21 January 2022.

**Catherine L. Mann**, Bank of England, London, UK.

**Lennart Brandt**, Bank of England, London, UK.

Figure 1
**Measures of UK inflation, % year-on-year**

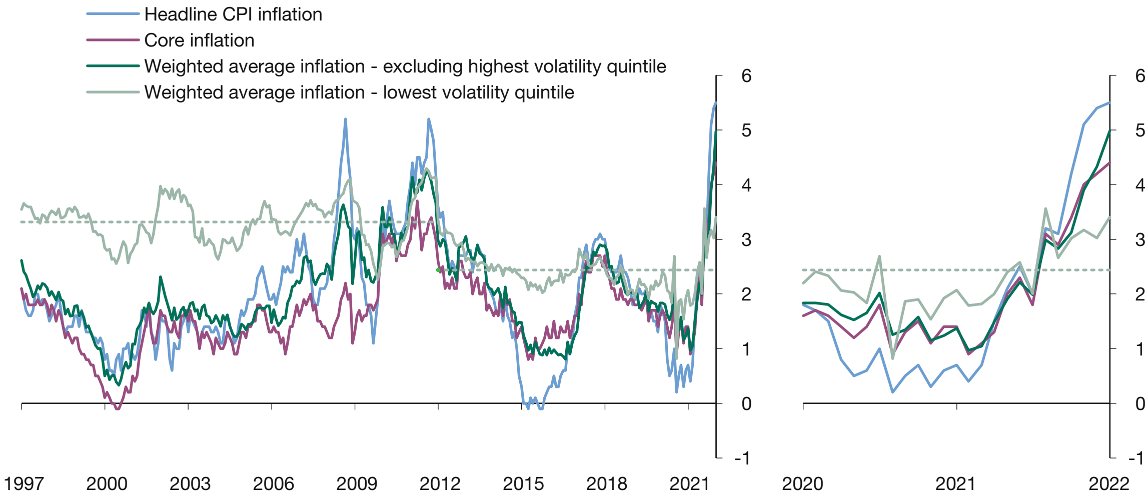

Notes: Average inflation by volatility is constructed by ranking the 85 class-level COICOP categories in the inflation basket by their realised volatility in the period 1997-2019 and then computing weighted average monthly year-on-year inflation within or excluding some portion of the basket. Latest observation: January 2022.

Sources: Office for National Statistics and authors' calculations.

noisy headline series. Examples of the former are various commonly watched "core" inflation rates, which typically strip out those components that are a priori considered too volatile to carry much of the long-term signal, such as the prices for energy and food. Examples of the latter range from simple pointwise means and medians of the monthly distribution of inflation rates – sometimes "trimmed" to exclude outliers within the month – to more complex statistical estimates extracted, for instance, using principal component analysis and dynamic factor models.[2]

While exclusion and inclusion of certain components is usually determined by convention or common knowledge, attempts have been made to inform the choice by statistical methods. For example, the ECB's "Supercore" series (O'Brien, 2018) isolates those components of the inflation basket that are estimated to be sensitive to economic slack. These components ought to be those most influenced by monetary policy based on a Phillips curve framework. On the other hand, the Atlanta Fed's "Sticky Price" index (Bryan & Meyer, 2010) attempts to single out components that change prices only very infrequently, i.e. those that are less sensitive to overall economic conditions.[3]

2 See for example the ECB's "Persistent and Common Component of Inflation" (Bańbura and Bobeica, 2020).

3 The selection of components for the "Sticky Price" index follows from a study of firm-product level price setting behaviour (Bils and Klenow, 2004).

The theme common to these measures is to remove the especially volatile component of inflation which is driven by perhaps large but in the end transitory shocks. The resulting series may then plausibly be considered a measure of underlying or trend inflation. The next section offers another attempt at stripping out the most volatile component by classifying components directly by their historical realised volatility.

## A volatility-based measure of inflation

For the simplest example (displayed in Figure 1), we compute and then aggregate into volatility buckets realised volatility of the 85 items in the Office of National Statistics' CPI basket over the period starting with the Bank's independence in 1997 and ending before the onset of the COVID-19 pandemic. We do not average across the pandemic period since the price-setting behaviour in a time of lockdowns and supply bottlenecks may be fundamentally different to what came before. However, we do find that the properties of the CPI and its components were largely consistent with the behaviour before the coronavirus pandemic – conditional on a deep recession and the subsequent recovery. Only from the second half of 2021 onwards do we see a marked difference in the aggregate behaviour of CPI inflation.

Alternatively, one might compute volatility separately for certain subsamples or on rolling windows. We find, however, that the relative ranking of components by volatility does not materially change in that case. The relative sensitivity of CPI components to different shocks, as measured by their higher moments, appears to be relatively stable even if their first moments can swing quite significantly.

As is well known, headline inflation has now surpassed its previous post-independence peaks, reaching 5.5% in January 2022. A large part of this increase is being driven by high and rising energy prices but even when adjusting for their direct impact, inflation rates are still well above the Monetary Policy Committee's (MPC) target. Figure 1 plots these series (headline and core CPI inflation) alongside two volatility-based measures of inflation.

The first series, which is the weighted average inflation excluding the most volatile fifth of components measures, as expected, is something close to core inflation. What is more surprising – and perhaps more worrying – is the behaviour of the lowest-volatility fifth of components. By definition, these components tend to adjust relatively little over time. Some examples are pharmaceutical products and hairdressing, but also housing rents, and restaurants and canteens. These latter two each account for over 8% in the CPI basket and are therefore important for the behaviour of the aggregate.

Note that the low volatility components do not anchor inflation to the target – indeed they run above the target for the whole period since 1997. But, inflation within this bucket has been confined to rather narrow and stationary bands around some mean for most of the last 25 years, with the mean apparently having shifted down by about one percentage point in the post-2011 period (see dotted line in Figure 1). More research is necessary to assess the cause of this step-down and is beyond the scope of this article. However, since inflation was – on average – at target both before and after 2011, the step-down in the lowest-volatility bucket must not have decisively driven aggregate inflation. It is tautological but the mixture of shocks and policy hitting both high- and low-volatility components was consistent with achieving the 2% inflation target in both regimes.

Starting in the second half of 2021, however, rates in the lowest-volatility bucket have left their range of the last decade and now look more in line with the period of 2011 and before. High inflation clearly is no longer limited to components that are typically quite volatile, but now has seeped into those that typically are rather stable. This raises a number of difficult questions for a monetary policymaker at the current juncture: Are these components just experiencing a pandemic-related jump to settle down soon? Or are cost-price dynamics that push the volatile components being embedded throughout. If robust inflation can be found in more than just isolated pockets, how will it get back to target? Surely, macroeconomic conditions exist that are consistent with achieving the inflation target while inflation in the low-volatility components is north of 3%. But that implies that there needs to be a drag, i.e. inflation below 2%, from other components.

## Strong for longer

Looking into and beyond 2022, there is a key role for expectations, which if realised could mean that inflation stays strong for longer. For firms, 2021 exposed them to significant cost-push factors including increasing costs of shipping and raw materials, export-related costs, rising wholesale energy prices and increasing wage pressures (arising from both staff shortages and underlying wage pressures such as minimum wage increases). In the Bank's DMP, some firms also mentioned higher costs associated with insurance, debt repayments, $CO_2$ emission reductions and coronavirus safety measures (Bank of England, 2022d). Will firms be able to pass these costs into their prices in 2022?

The DMP shows an asymmetry in the relationship between prices and sales. Firms that experienced faster sales increases due to COVID-19 also hiked their prices at much steeper rates than firms reduced their prices as their sales fell.[4] This convex price profile using firm-level data is mimicked in research using macro data that finds a convex Phillips curve relating inflation to slack in the economy (Collins et al., 2021).

The MPC's November 2021 Monetary Policy Report (Bank of England, 2021) recognised that downward price rigidity is an upside risk to the inflation outlook as the near-term effects of COVID-19 pandemic fall away. Firms' pricing expectations from the DMP survey solidify this upside risk for 2022. Similarly for wages, Bank research shows wage demand and inflation expectations are correlated, and that items that consumers buy frequently, such as energy, food and clothing have particular salience for their short-term perceptions of inflation (Bonciani et al., 2022). Given the rapid increase in prices for some of these salient items, it is not surprising that consumer expectations for inflation in the short term have jumped, too. Wage compression has been a feature of the period after the global financial crisis, but the environment of higher price inflation and tighter labour markets may herald a regime change for wage outturns.

4 See Chart 2.27 in the November 2021 Monetary Policy Report (Bank of England, 2021).

Figure 2
**Scenarios for average weekly earnings**

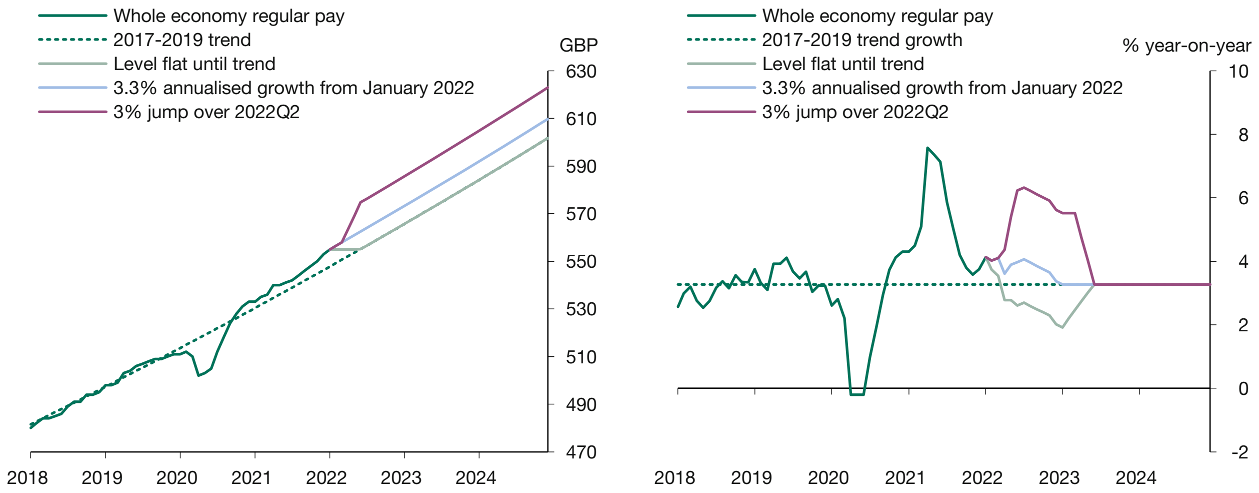

Note: Latest observation: January 2022.

Sources: Office for National Statistics and authors' calculations.

## Scenarios for inflation based on alternative historical outturns and expectations

The earlier section showed that the behaviour of inflation within different buckets of the volatility distribution could be revealing a fundamentally changed macroeconomic environment. The surveys implied that current price and wage momentum is being reflected in 2022 wage and price expectations, which suggests an embedded dynamic. Naturally, this is speculative and we will continue to learn more from macro data and micro surveys as we go along. However, even assuming that we are not on the cusp of a regime change and that the mixture of shocks in the economy will return to what it was before the COVID-19 pandemic, we are still faced with the possibility of staying in uncomfortable territory with inflation above target for longer than initially thought.

This section shows some simple arithmetic exercises about how wages and goods prices might evolve going forward if some of the 2021 wage and price increases are repeated in 2022, as has been suggested by the Bank of England's surveys and agents' intelligence.

Consider wages first. As Figure 2 shows, wages have rebounded from their 2020 trough, leading to high year-on-year outturns in 2021. As of January, average wages were slightly elevated compared to their pre-coronavirus trend but showed little sign of spiralling. For the arithmetic exercise, to project forward from the current data, we make three very simple assumptions: One scenario holds wages fixed until they reach their pre-pandemic trend. A second continues the historical trend from the latest data point. A third scenario shows what would happen to wages if there was another strong settlement season in 2022. According to the Bank's agents, some further upward pressure on wages is to be expected in the coming months as firms and workers adjust to the higher costs of doing business and costs of living (Bank of England, 2022a). To stand in for such a scenario, we let average weekly pay rise by 3% over the second quarter, then return to its pre-COVID-19 trend growth. From the perspective of wage inflation, wage settlements even only as strong as last year would keep wage inflation strong for longer.

Ultimately, however, it is firms' pricing decisions that generate inflation. The next set of charts explores what would happen if the goods price increases of 2021 were repeated in 2022, as surveys suggest firms will attempt to do. Over the course of last year, core goods prices had already risen markedly and now stand about 4% above their trend level (Figure 3, left-hand panel). They have come off the top a little bit in January but are obviously still well above trend. If in fact this decrease in goods prices continues, so much the better for inflation rates and household purchasing power in the near term. After all, any decrease in goods prices is directly deflationary. For now though, similar to the wages example above, we show three scenarios for goods prices where levels at least are sustained. The only difference to wages is that,

Figure 3
**Scenarios for core goods prices**

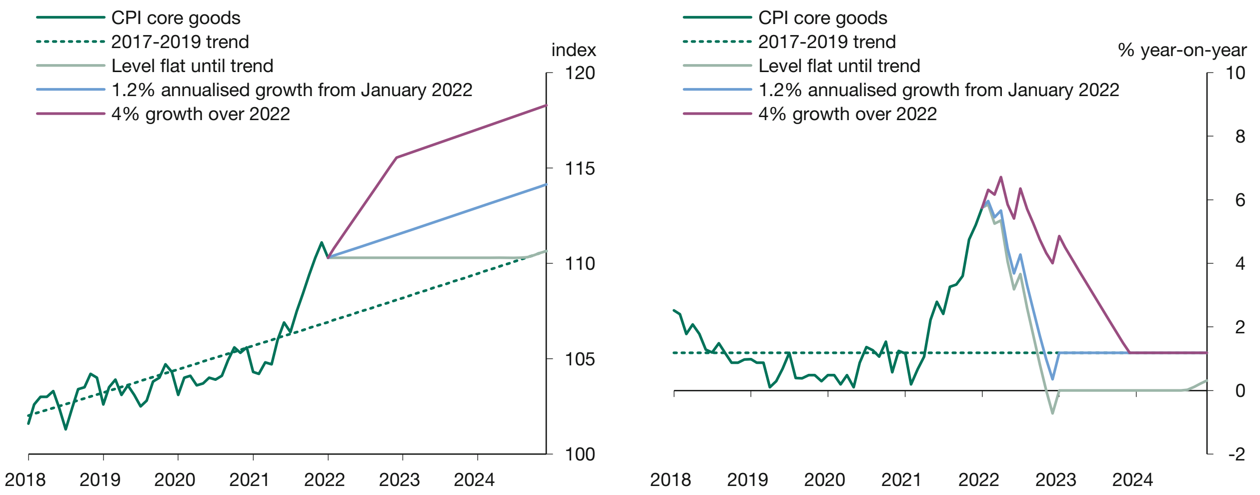

Note: Latest observation: January 2022.

Sources: Office for National Statistics and authors' calculations.

in the upside scenario, we assume a 4% rise in prices over the whole of 2022, consistent with the outturn for 2021.

From the right hand panel it is immediately apparent that all of these pricing scenarios imply robust goods price inflation rates by year-on-year metrics. If we take the current level of prices as given, most inflation in the near term is already baked in. Even if prices stopped rising right now, goods inflation would arithmetically increase to over 6% in February. Peak goods inflation does not differ much between scenarios, either: It is going to be between 6% and 8%, and sometime in the first half of this year. What does differ significantly is the length of time for the price shock to work through and return inflation to trend.

In the two more benign scenarios – where goods prices are unchanged or only grow at their historical annual average of 1.2% – core goods inflation reaches its pre-COVID-19 average by the end of the year. But in the scenario where firms are able to push through another 4% price increase, inflation remains elevated through all of 2023. From a mechanical perspective, any shocks to the price level only wash out after one full year has passed. This exercise assumes that the jump in prices is not repeated in 2023 or beyond – i.e. that cost inflation does not get embedded in pricing behaviour.

There are a variety of underlying factors which might push up prices in 2022. Uncertainty is one of them: Chan (2021) finds that, in a model with incomplete information, beliefs about competitors' pricing can affect the optimal pricing behaviour of the firm, and thereby affect aggregate outcomes. A loosening of supply constraints, perhaps paradoxically, could also support faster price growth in the short run as loosening supply is met with even stronger demand.[5]

Global trade issues seem more set to push up prices. Although shipping rates look like they may have peaked, they are still very high compared to their history, raising the cost of trade around the world. UK Purchasing Managers' Indices reflect this ongoing disruption. While variation in delivery times generally has small effects on aggregate prices, the especially severe disruptions in 2021, which are likely to persist, will likely show up in prices into 2022.

Further, the UK's evolving trading situation post-Brexit may exacerbate any inflationary impulse from global goods and commodity markets by adding another wedge of administrative costs as well as changing the competitive landscape and perhaps altering the variety of products available. Bank research estimates a widening Brexit wedge on the supply side of the economy of some 2% by the end of 2024 from the pre-pandemic trend. Already,

5 See for example Cesa-Bianchi and Ferrero (2021) who, in US data, find evidence for complementarities at the product level through which sectoral shocks can cause aggregate fluctuations.

both UK export and import volumes have been tracking well below their G7 peers.

Finally, not discussed here, but extremely important for the near-term dynamics of inflation is the impact of energy prices and the revision of the Ofgem price cap. The Russian invasion of Ukraine has already caused another shock to energy prices and – given the lagged calculation of the cap – will likely have sizeable effects on consumer prices beyond the current year.[6]

## Implications for monetary policy

Waves of surging inflation – from the reopening from COVID-19 and associated energy and goods-price inflation, from the Russian invasion of Ukraine and further leg-up in energy prices, and likely from the most recent coronavirus lockdowns of production facilities in China – have pushed core and headline inflation in many advanced economies to highs not seen for many decades. What was transitory at first has spread to more product categories and into labour markets, raising even the least volatile components of the UK CPI, perhaps heralding a regime change to where prices in these categories once again trend above the inflation target.

Current price and wage expectations coming from the DMP survey are inconsistent with the Bank of England's 2% target and, if they are realised in 2022, are likely to keep inflation strong for longer, which could embed a reinforcing cost-price dynamic. The longer wages and prices stay above target, the more persistent the headline inflation. A first defence against persistence is to lean against expectations. However, expectations are not the only factor relevant for monetary policy. The price increases already in train and those embedded via the Ofgem price cap will hit household income, and likely will constrain purchasing power and therefore pricing power over non-energy goods and services. In considering the appropriate policy path to achieve the inflation target in the medium term, it is necessary to evaluate the tenacity of wage and price expecttions against expected aggregate demand outcomes.

6 For more information on the effects of energy price shocks on the UK economy, see the scenarios using standard forecast methodology versus the full futures curves alternative in the November and February Monetary Policy Reports (Bank of England, 2021 and 2022c).
